# Opioid Exposure Induces the Expression of the Purinergic Receptor *P2RY11* in Human Nociceptors

**DOI:** 10.3390/cells15161464

**Published:** 2026-08-15

**Authors:** Jenna B. Demeter, Sean D. McNally, Maddy R. Koch, Sherwin Thiyagarajan, Jeanette A. Montoya, Liliana Vega, Paul Abboud, Sascha R. A. Alles, Reza Ehsanian, June Bryan I. de la Peña

**Affiliations:** 1 Translational Pain Group, Department of Anesthesiology and Critical Care Medicine, University of New Mexico Health Sciences Center, Albuquerque, NM 87131, USAsmcnally@salud.unm.edu (S.D.M.); mrkoch@salud.unm.edu (M.R.K.); shthiyagarajan@salud.unm.edu (S.T.); jeamontoya@salud.unm.edu (J.A.M.); livega@salud.unm.edu (L.V.); pabboud@salud.unm.edu (P.A.); rehsanian@salud.unm.edu (R.E.); 2 Department of Anesthesia, Cincinnati Children’s Hospital Medical Center, Cincinnati, OH 45229, USA; sascha.alles@cchmc.org

**Keywords:** metabotropic purinoceptor, P2Y, hDRG, pain, longitudinal RNA-seq, clustering analysis

## Abstract

**Highlights:**

**What are the main findings?**

- Time-series RNA sequencing of human iPSC-derived nociceptors identified *P2RY11* as a robust and sustained morphine-inducible transcript.
- *P2RY11*/P2Y11 was detected in human dorsal root ganglion neurons, including peripherin- and TRPV1-positive nociceptor populations, and P2Y11 immunoreactivity increased after morphine exposure.

**What are the implications of the main findings?**

- These findings identify *P2RY11*/P2Y11 as a previously underappreciated, morphine-responsive target in human nociceptors that may contribute to opioid-driven peripheral plasticity and requires functional testing.
- Because *P2RY11* lacks a mouse/rat ortholog, this work highlights the importance of human-based models for discovering opioid-responsive pain mechanisms not captured by standard rodent systems.

**Abstract:**

Primary sensory neurons of the dorsal root ganglion (DRG) express mu-type opioid receptors and undergo plasticity that can contribute to both analgesia and maladaptive outcomes such as opioid tolerance and opioid-induced hyperalgesia. Most mechanistic work has relied on rodent models, which may not fully capture the repertoire of opioid-responsive pathways present in humans. In this study, we tested whether morphine exposure reshapes gene expression programs in human nociceptors. Human induced pluripotent stem cell (hiPSC)-derived nociceptors were exposed to morphine (3.5 μM) acutely (1h, 16h) or repeatedly (2–3 days; daily 16h exposure separated by 8h washout) and profiled by time-series RNA sequencing. The transcriptional response to morphine included the induction of a small set of genes across exposure paradigms. *P2RY11*, encoding the purinergic G protein-coupled receptor P2Y11, was the most robustly induced transcript across the time course, and genes involved in purinergic signaling pathways exhibited coordinated expression dynamics. Since *P2RY11* lacks a mouse/rat ortholog, its contribution to nociceptor biology and opioid responses has remained largely underexplored. Using human DRG tissue and primary human DRG cultures from organ donors, we detected *P2RY11* mRNA and P2Y11 protein in neuronal populations. We observed increased P2Y11 expression in peripherin-positive neurons after morphine exposure in vitro, corroborating our sequencing results. These findings identify *P2RY11*/P2Y11 as a morphine-responsive purinergic receptor in DRG neurons and nominate purinergic signaling as a candidate pathway contributing to opioid-driven peripheral plasticity.

## 1. Introduction

Opioid analgesics are among the most effective medications for moderate to severe pain management. However, their long-term use is constrained by adverse outcomes, including opioid tolerance and opioid-induced hyperalgesia (OIH) [1]. Opioid tolerance reflects a progressive loss of analgesic efficacy that can drive dose escalation, whereas OIH is a paradoxical increase in pain sensitivity that can emerge during opioid exposure [2,3]. These adaptations complicate pain management and motivate efforts to identify molecular mechanisms and targets that separate sustained analgesia from maladaptive plasticity.

Primary sensory neurons of the dorsal root ganglion (DRG) express the mu-type opioid receptor (MOR-1) across species, including in humans, and are direct targets of opioids [4]. In addition to mediating peripheral opioid analgesia, DRG neurons undergo opioid-driven plasticity that can alter excitability, intracellular signaling, and responsiveness to inflammatory mediators [5]. However, most mechanistic insight into these processes comes from rodent models. Comparative transcriptomic studies demonstrate substantial divergence between human and rodent DRG, particularly among G protein-coupled receptors (GPCRs) and other druggable signaling proteins [6,7,8]. These differences raise the possibility that opioids engage pathways relevant to human pain that are not necessarily represented in non-human preclinical models. This has driven the use of human in vitro platforms, including human induced pluripotent stem cell (hiPSC)-derived nociceptors and primary human DRG (hDRG) neurons from organ donors. These systems recapitulate key molecular and functional features of human DRG neurons [9,10,11] and enable controlled opioid exposure experiments in a human genetic context.

Purinergic signaling is an important modulator of nociceptor function linked to pain processing [12]. Extracellular nucleotides such as ATP—released during tissue injury, inflammation, or neuronal activity—activate P2X ligand-gated ion channels and P2Y GPCRs expressed in neurons and non-neuronal cells along the pain axis [13,14]. P2Y receptors have been implicated in nociceptor sensitization and pain transmission. Rodent pharmacology studies at peripheral and spinal sites have reported contributions of P2Y1, P2Y6, and “P2Y11-like” signaling to inflammatory and neuropathic pain, indicating that P2Y-family GPCRs can modulate nociceptor plasticity [13,15,16]. Purinergic pathways also intersect with opioid adaptations. For example, microglial purinergic signaling and P2X4 and P2X7 receptors have been linked to opioid tolerance and OIH in rodents [17,18,19]. Within the P2Y family, P2Y purinoceptor 11 (P2Y11)—encoded by *P2RY11*—is distinctive because it couples to both Gq- and Gs-dependent signaling. This positions it to regulate intracellular calcium and cAMP pathways that influence neuronal excitability and transcriptional plasticity [20,21,22]. Despite these features, P2Y11 has been minimally explored in pain biology, in part because *P2RY11* lacks an ortholog in mice and rats, which complicates genetic and pharmacologic studies in conventional models [20]. Interpretation of previous rodent findings involving a “P2Y11-like” receptor is limited by the absence of a true *P2RY11* gene and by ligand selectivity across P2Y receptors, underscoring the need to examine P2Y11 directly in human sensory neurons [20]. Recent transcriptomic resources found that *P2RY11* is expressed in hDRG neurons [23]. However, *P2RY11* has not been studied in human nociceptors, including its upstream regulators, downstream signaling cascades, and functional consequences.

In the present study, we used human-based models to define how morphine reshapes gene expression programs across acute and repeated exposure. hiPSC-derived nociceptors were treated with morphine (3.5 μM) under single-exposure (1h, 16h) and repeated-exposure (2–3d; daily 16h exposure separated by 8h washout) conditions and profiled by time-series RNA sequencing. Differential expression and longitudinal clustering analyses revealed morphine-responsive transcriptional dynamics. This included robust and sustained induction of *P2RY11* and coordinated modulation of gene expression related to purinergic and P2Y11 signaling. We then validated *P2RY11* and P2Y11 expression in hDRG tissue and primary hDRG cultures from organ donors. We detected P2Y11 protein in peripherin- and TRPV1-positive nociceptors and observed increased P2Y11 immunoreactivity in morphine-treated hDRG cultures, corroborating our sequencing results. Together, these findings identify *P2RY11* as an opioid-responsive purinergic receptor in DRG neurons and provide a rationale for testing whether P2Y11 signaling contributes to opioid-driven peripheral plasticity.

## 2. Materials and Methods

### 2.1. hiPSC-Derived Nociceptor Culture

hiPSC-derived nociceptors (#1020-1M, lot #SN204628, RealDRG™, Anatomic Incorporated, Minneapolis, MN, USA, female donor) were thawed and cultured according to the manufacturer’s recommendations. First, 12-mm glass coverslips (for immunocytochemistry) or 12-well plates (for RNA extraction) were pre-coated with poly-L-ornithine (#A-004-C, MilliporeSigma, Burlington, MA, USA) followed by recombinant human laminin-511 E8 fragment protein (iMatrix-511, #AMS.892, AMSBIO, Abingdon, UK). Thawed and pelleted cells were resuspended in Senso Maturation Medium (#1030, Anatomic) supplemented with 1% antibiotic–antimycotic (#A5955, MilliporeSigma, Burlington, MA, USA) and seeded at a density of approximately 25,000 cells per cm^2^. Cultures were maintained at 37 °C in a humidified incubator with 5% CO_2_. 80% media exchanges were performed every other day. Cells were allowed to mature for 14 days prior to experimental manipulation.

### 2.2. Morphine Exposure Paradigm

To model sustained opioid receptor engagement in a controlled human nociceptor system, hiPSC-derived nociceptors were treated with 3.5 μM morphine in culture medium. Morphine sulfate (Infumorph 500, NDC 0641-6040-01, Hikma Pharmaceuticals USA Inc., Berkeley Heights, NJ, USA) was obtained through approved institutional channels and handled in accordance with the Drug Enforcement Administration (DEA) controlled-substance regulations. Working solutions were prepared by diluting into culture medium to a final concentration of 3.5 μM. Experimental groups included baseline (0h), acute exposure (1h), prolonged single exposure (16h), and repeated exposure (2d and 3d). Repeated exposure consisted of 16h morphine treatment followed by an 8h washout in morphine-free medium, repeated across 2–3 days. Washout was initiated by aspirating morphine-containing medium and replacing it with fresh morphine-free medium. All untreated wells also received fresh media at the washout points. At each treatment time point, morphine was added to existing media by transferring half of the media to a microfuge tube, adding an appropriate volume of morphine sulfate, and returning the morphine-containing media to the well. As a control, this process was additionally repeated for untreated wells, except that no morphine was added. The 3d time-point samples were treated first and the 1h time-point samples last such that all samples were simultaneously collected at the end of their respective treatment durations on days in vitro (DIV) 17. Sample collection at 2d and 3d occurred at the end of the second and third 16h morphine exposures, respectively. The number of biological replicates (independent culture wells) per condition was *n* = 3 per time point.

The 3.5 μM concentration was selected as a mechanistically informed in vitro exposure paradigm based on the literature. In primary sensory neurons, morphine exposures in the low- to mid-micromolar range (1–10 μM) over hours to days induce adaptive changes linked to sensitization and an opioid tolerance-like phenotype [24,25,26]. In addition, 10–20 μM morphine over several days has been shown to drive MOR-1-dependent MAPK and CREB signaling [27]. Notably, a similar paradigm of 3.3 μM morphine for 5 days has been used in cultured DRG neurons to elicit transcriptional changes [28]. This places our 3.5 μM exposure in the lower middle of this established in vitro range. We incorporated both early (1h) and sustained (16h) time points as well as repeated exposure with an intervening drug-free interval to capture adaptive programs that can emerge with prolonged receptor activation and/or drug removal.

### 2.3. Sample Collection, RNA Extraction, Quality Control, Library Preparation, and Sequencing

For transcriptomic profiling, all groups were processed using identical handling and timing. All samples were simultaneously collected at the conclusion of the treatment paradigm, preserved in DNA/RNA Protection Reagent (New England Biolabs, Ipswich, MA, USA), then immediately processed for RNA extraction. Total RNA was extracted using the Monarch Total RNA Miniprep Kit (New England Biolabs, Ipswich, MA, USA) according to the manufacturer’s instructions. RNA yield and purity were evaluated by spectrophotometry (NanoDrop, Thermo Fisher Scientific, Waltham, MA, USA), and samples meeting standard thresholds (OD 260/280 ≥ 2.0 and OD 260/230 ≥ 1.8) were advanced for further analysis. RNA integrity was assessed using an Agilent 5400 Fragment Analyzer (Agilent Technologies, Santa Clara, CA, USA), and only samples with high integrity (RIN ≥ 7.5) were used for library preparation. mRNA libraries were prepared by Novogene (Sacramento, CA, USA) using an oligo-dT enrichment-based eukaryotic mRNA library preparation protocol, followed by paired-end sequencing (150 bp) on an Illumina NovaSeq X Plus platform. Sequencing yielded ≥ 6 Gb of coverage, corresponding to ≥20 million read pairs per sample. Samples were processed for bulk RNA-seq as hiPSC-derived nociceptor cultures are highly homogeneous, providing high sensitivity for detecting treatment-responsive transcriptional changes and supporting robust time-course differential expression and clustering analyses.

### 2.4. RNA-Seq Processing and Differential Expression Analysis

Raw reads were processed using an RNA-seq pipeline ranked among the best for accurate identification of differentially expressed genes (DEGs) [29]. Quality assessment was performed with FastQC. Reads were aligned to the Ensembl human reference genome GRCh38 (Ensembl release 113) using HISAT2 [30]. Quantification was performed using StringTie [31], and Ensembl annotations were added from biomaRt [32,33]. Pairwise differential expression was performed using the quantile-adjusted conditional maximum likelihood (qCML) method in edgeR [34]; each morphine-treated sample (1h, 16h, 2d, 3d) was compared to untreated baseline samples (0h). *p*-values were adjusted for multiple testing using the default Benjamini–Hochberg false discovery rate (FDR) approach. Genes with an adjusted *p*-value (*p*-adj) ≤ 0.05 and |log2FC| ≥ 0.6 were considered DEGs. The heatmap of all DEGs across time points was generated using pheatmap, using log2TPM as input and incorporating hierarchical clustering and scaled intra-gene expression across samples. Gene set enrichment analysis (GSEA) using Gene Ontology (GO) terms was conducted using clusterProfiler [35], with the edgeR results ranked by log2FC for each time point relative to baseline as the input and a significance threshold of *p*-adj ≤ 0.05.

### 2.5. Time-Series Differential Expression, Clustering, and Functional Enrichment Analyses

To clearly identify genes exhibiting differential expression over the time course when taken as a whole, we utilized ImpulseDE2 [36]. Genes with *p*-adj ≤ 0.05 were considered significant dynamic differentially expressed genes (dDEGs). To identify coordinated transcriptional responses to opioid exposure over time, genes exhibiting temporal variation (*p* ≤ 0.05) were grouped using time-series clustering analysis implemented in TCSeq, with transcripts partitioned into six distinct expression clusters (c = 6). Clustering was performed based on similarities in normalized and scaled expression trajectories across treatment time points using a fuzzy c-means algorithm, allowing visualization of dynamic cluster-specific expression patterns over the course of exposure. Functional annotation of each cluster was conducted using GO over-representation analysis (ORA) in clusterProfiler [35], using all genes expressed in the dataset as the background set and a threshold of *p* ≤ 0.05 for term inclusion. Biological processes relevant to G protein-coupled receptor signaling, purinergic signaling, ion transport, and calcium homeostasis were emphasized for downstream interpretation due to their relevance to nociceptor function, opioid signaling, and mechanisms of key DEGs/dDEGs.

### 2.6. Human Dorsal Root Ganglion (hDRG) Tissue Acquisition

Human dorsal root ganglion (hDRG) tissue was obtained from ethically consented organ donors at the University of New Mexico in collaboration with New Mexico Donor Services. All procedures were conducted in compliance with Institutional Review Board (IRB) guidelines and were approved by the University of New Mexico Health Sciences Center Human Research Review Committee (IRB approval numbers 23-205 and 25-407). Donor demographics, medical information including opioid toxicology, and allocation of samples to hDRG tissue immunostaining and primary-culture experiments are summarized in Supplemental Table S1. For all donors, DRGs were recovered within 4h of aortic cross-clamp, and tissue was either (i) embedded and frozen immediately for immunohistochemistry or (ii) temporarily incubated in Hibernate A before dissociation and primary culture. Detailed outpatient medication records, analgesic prescriptions, and pain histories were not released to research teams by the organ procurement organization and are therefore unavailable.

### 2.7. Primary hDRG Neuron Culture

Primary hDRG neurons were prepared from ethically consented organ donor tissue as previously described [37,38]. Briefly, DRGs were incubated for a few hours in Hibernate A (BrainBits, Springfield, IL, USA). Then tissue was manually dissected and enzymatically dissociated using STEMxyme I (#LS004106, Worthington Biochemical Corporation, Lakewood, NJ, USA) and DNase I (#LS002193, Worthington Biochemical Corporation, Lakewood, NJ, USA) at 37 °C using a shaking water bath interspersed with gentle trituration. Cells were filtered and then purified using a bovine serum albumin (BSA) density gradient. The cell pellet was resuspended in medium based in Neurobasal A (#10888022, Gibco, Thermo Fisher Scientific, Waltham, MA, USA) and supplemented with N2 (#07152, STEMCELL Technologies, Vancouver, BC, Canada), SM1 (#05711, STEMCELL Technologies, Vancouver, BC, Canada), GlutaMAX (#35050061, Thermo Fisher Scientific, Waltham, MA, USA), antibiotic–antimycotic solution (#A5955, MilliporeSigma, Burlington, MA, USA), nerve growth factor (NGF; #32061, Cayman Chemical, Ann Arbor, MI, USA; final concentration 10 ng/mL), 5-fluoro-2′-deoxyuridine (#sc-202425, Santa Cruz Biotechnology, Dallas, TX, USA), and uridine (#A15227.06, Thermo Fisher Scientific, Waltham, MA, USA). Cells were then plated on coverslips coated with poly-D-lysine (PDL, #A-003-E, MilliporeSigma, Burlington, MA, USA). Cultures were maintained at 37 °C in 5% CO_2_ with partial media exchanges every 2–3 days. Neurons used for ICC were treated with 3.5 μM morphine for 1h, 16h, 2d, or 3d without washout or were left untreated, and all samples were simultaneously fixed at the end of the time course. A continuous morphine exposure without washout was used for the primary hDRG cultures because repeated washout cycles matching the hiPSC paradigm were poorly tolerated by these sensitive primary neurons and compromised culture integrity. The continuous paradigm therefore provides a convergent, independent test of morphine-induced P2Y11 induction rather than a replication of the identical hiPSC regimen.

### 2.8. Immunohistochemistry (IHC) of hDRG Tissue: Processing and Cryosectioning

For immunostaining, fresh hDRG tissue from donor H27 (Supplemental Table S1) was embedded in OCT and placed immediately at −80 °C. Cryosections were cut at 18-μm thickness, mounted onto slides, and fixed in 4% paraformaldehyde (PFA) for 15 min at room temperature prior to staining.

### 2.9. Immunocytochemistry (ICC) of Cultured hiPSC-Derived Nociceptors and Primary hDRG Neurons

hiPSC-derived nociceptors or hDRG cells from three organ donors—H27, H30, and H34 (Supplemental Table S1)—were cultured on 12-mm glass coverslips. Upon sample collection, slips were washed with PBS and then fixed in 4% PFA for a total of 15 min (3 × 5-min), with PBS washes interspersed between fixative changes. Cells were then permeabilized with 0.5% Triton X-100 (#A16046.AE, Thermo Fisher Scientific, Waltham, MA, USA) in PBS (PBST) for 5 min at room temperature, followed by 3 × 5-min PBS washes. Coverslips were then blocked for 1h at room temperature in blocking buffer consisting of 5% normal goat serum (NGS; #0060-01, SouthernBiotech, Birmingham, AL, USA) in PBST. Coverslips were incubated overnight at 4 °C in a humidified chamber with primary antibodies diluted in 1% NGS buffer. Antibodies included chicken anti-peripherin (#PA1-10012, Thermo Fisher Scientific, Waltham, MA, USA, 1:5000) and either rabbit anti-MOR-1 (for hiPSC-derived nociceptors; #RA10104, Neuromics, Edina, MN, USA; 1:1000) or rabbit anti-P2Y11 (for hDRG cultures; #NLS867, lot #42179, Novus Biologicals, Centennial, CO, USA; 1:100). The next day, coverslips were washed 3 × 5 min in PBST and incubated for 1h at room temperature in the dark with species-appropriate fluorophore-conjugated secondary antibodies. Secondary antibodies were diluted in 1% BSA buffer and included goat anti-chicken (#A-11039, Thermo Fisher Scientific, Waltham, MA, USA; 1:1000) and goat anti-rabbit (#A-32731, Thermo Fisher Scientific, Waltham, MA, USA; 1:1000). Coverslips were washed an additional 3 × 5 min with PBS and mounted using Fluoromount-G with DAPI (#00-4959-52, Thermo Fisher Scientific, Waltham, MA, USA). Slides were cured and sealed for imaging. For P2Y11 and peripherin immunostaining, specificity was assessed using no-primary, secondary-only controls, which yielded negligible signal (Supplemental Figure S2).

### 2.10. Confocal Imaging and Quantitative Fluorescence Analysis

Immunocytochemical imaging was completed using a Zeiss LSM 900 confocal microscope (Carl Zeiss Microscopy GmbH, Jena, Germany), and quantitative fluorescence analysis was performed using Fiji (ImageJ v2.18.0/1.54p). Individual neurons and neuron clusters captured in the images were manually outlined using the freehand selection tool and marked as regions of interest (ROIs). Three background ROIs were also taken in each image from different regions. Cell and background ROIs were first obtained in the peripherin channel. These ROIs were then used for measurements in the MOR-1 or P2Y11 channels. Mean grey value (mean intensity of pixels within the ROI) was adjusted for the target of interest by subtracting the average of the background mean grey values. For MOR-1 immunoreactivity in hiPSC-derived nociceptors, mean grey value from each neuron ROI was normalized to the 0h baseline, and statistical analysis was completed using Prism v10.5.0 (GraphPad Software, San Diego, CA, USA); following normality tests, data were analyzed using a Kruskal–Wallis one-way ANOVA followed by post-hoc analysis with Dunn’s multiple comparisons correction.

For P2Y11 intensity in primary hDRG neurons, immunoreactivity was also quantified as background-adjusted mean grey value. Morphine was applied at the level of the culture/coverslip. Quantification comprised 3 organ donors, 14 cultures/coverslips, 129 imaging fields, and 239 neuronal ROIs, with counts at each level given in Supplemental Table S1. Images were quantified using coded identifiers, with the experimenter blinded to exposure condition. To account for the hierarchical sampling structure of the per-neuronal-ROI data (donor > culture/coverslip > imaging field), adjusted mean grey values were analyzed along the time course using a linear mixed-effects model (lmerTest v3.2.1), with morphine exposure duration as the fixed effect and donor, culture/coverslip, and imaging field as nested random effects. The model was fit by restricted maximum likelihood. Because baseline P2Y11 intensity differed severalfold across donors/ imaging context and variance scaled with the mean, models were fit to log2-transformed adjusted mean grey values. Estimated marginal means at each time point were computed using Kenward-Roger approximation and back-transformed from the log2 scale. Fold-changes relative to 0h, 95% confidence intervals, and Dunnett-adjusted *p*-values were obtained for pairwise contrasts of each time point against 0h (emmeans v2.0.4). Neuronal ROI values are displayed normalized to the mean 0h intensity of the respective donor and plotted with donor-specific symbols. Fold-change values (means relative to 0h) and 95% confidence intervals are derived from the mixed-effects analysis; thus, the estimated marginal means are not identical to the arithmetic means of the plotted ROI values. We acknowledge that immunofluorescence intensity is a semiquantitative proxy for expression that depends on antibody, fixation, staining, and imaging conditions, and we interpret these measurements accordingly.

### 2.11. hDRG Single-Nucleus Transcriptomics Analysis

To explore *P2RY11* expression across hDRG sensory neuron subtypes, we utilized the Harmonized cross-species DRG reference atlas (v1), which contains integrated single-cell and single-nucleus RNA-seq data from 23 datasets across several rodent and primate species [39]. The atlas can be accessed at the following URL: https://painseq.shinyapps.io/harmonized_painseq_v1/ (accessed on 22 September 2025). Briefly, clustering was performed based on the expression of 2000 variable genes, and DRG neuron subtypes were classified by marker gene expression as provided in the atlas. We specifically employed data from three single-nucleus RNA-seq datasets from human DRG to determine the average *P2RY11* expression per cell and the percentage of hDRG neurons expressing *P2RY11*. Results were visualized using UMAP embeddings and neuron subtype-level bar plots.

## 3. Results

### 3.1. Time-Series RNA Sequencing Identifies Morphine-Regulated Transcripts

**Figure 1:**
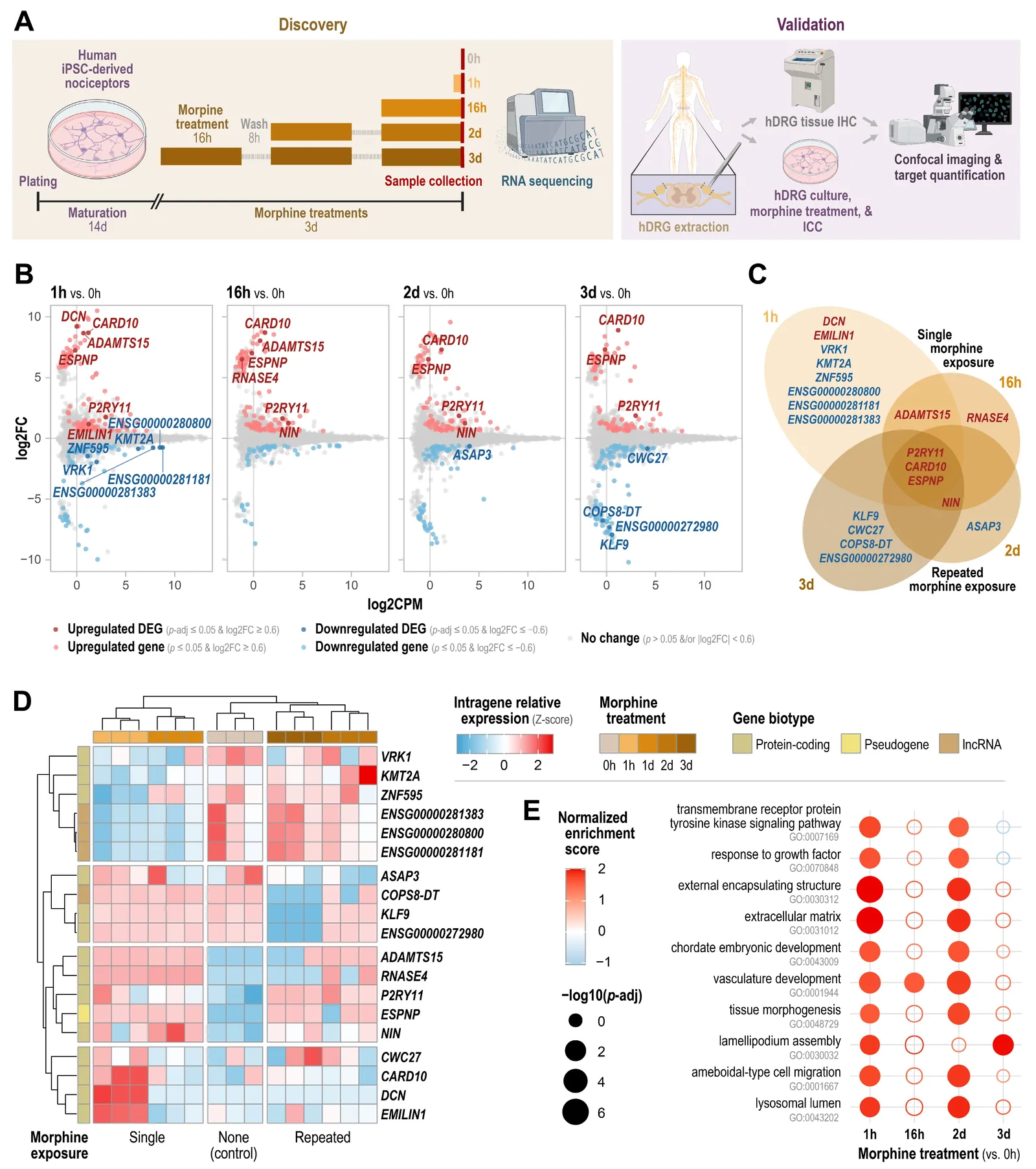
Time-series RNA sequencing identifies morphine-regulated transcripts. (**A**) Schematic of the experimental workflow. For transcriptomic discovery, hiPSC-derived nociceptors were matured for 14d, exposed to morphine (3.5 μM) acutely (1h, 16h) or repeatedly (2d, 3d)—consisting of daily 16h morphine treatments separated by 8h washout periods—and collected for RNA extraction and sequencing. A candidate target was then validated in human DRG (hDRG) tissue by immunohistochemistry (IHC) and in primary hDRG cultures by morphine treatment and immunocytochemistry (ICC), with both approaches culminating in confocal imaging with target quantification. Panel partially created in BioRender.com. (**B**) Volcano plots showing differential expression at each morphine time point relative to baseline (0h). Plots show log2 fold-change (log2FC) versus mean expression (log2CPM). Genes meeting DEG thresholds (*p*-adj ≤ 0.05 and |log2FC| ≥ 0.6) are highlighted (dark red, upregulated; dark blue, downregulated). Genes meeting a looser threshold (*p* ≤ 0.05 and |log2FC| ≥ 0.6) are shown in lighter hues. (**C**) Venn diagram summarizing overlap of DEGs across time points and exposure paradigms (single exposure: 1h and 16h; repeated exposure: 2d and 3d). Gene labels are colored by directionality (red, upregulated; blue, downregulated). (**D**) Heatmap of scaled intragene relative expression (Z-score) of DEGs across samples and morphine time points (0h, 1h, 16h, 2d, 3d), with hierarchical clustering of genes and samples. Annotation bars indicate exposure grouping and gene biotype. (**E**) Select Gene Ontology (GO) biological process terms enriched in each time-point comparison versus baseline by gene set enrichment analysis (GSEA). Dot size reflects significance (−log10[*p*-adj]), and dot color reflects normalized enrichment score. Open circles indicate terms not meeting the significance threshold.

To model opioid-driven transcriptional changes in a human nociceptor context, we exposed hiPSC-derived nociceptors to morphine (3.5 μM) using a time-course paradigm with sample collection at baseline (0h), after acute single exposure (1h), after sustained single exposure (16h), or after repeated exposure (2d or 3d; daily 16h morphine treatment followed by an 8h washout in morphine-free medium) (Figure 1A). Immunocytochemistry confirmed that hiPSC-derived nociceptors were peripherin-positive and displayed MOR-1 immunoreactivity across all time points (Supplemental Figure S1). This is consistent with reports that hiPSC-derived nociceptors develop functional mu-type opioid receptor signaling in vitro [40] and with electrophysiological characterization of hiPSC-derived sensory neurons from this same commercial source alongside human DRG neurons [11]. We next performed time-series RNA-seq to define morphine-responsive gene expression programs across exposure durations. Differential expression analysis at each time point relative to baseline revealed a small set of differentially expressed genes (DEGs) (*p*-adj ≤ 0.05, |log2FC| ≥ 0.6) (Figure 1B, Supplemental Table S2). Of all time points, the greatest number of DEGs (*n* = 12) were observed at 1h, suggesting a relatively quick acute transcriptomic response to morphine (Figure 1B). To highlight the expression patterns of genes altered by single and/or repeated morphine exposure, we compared their directionality and the time points at which each DEG appeared. In total, 8 DEGs were upregulated while 11 DEGs were downregulated (Figure 1C). While 14 genes appeared at singular time points, including all downregulated DEGs, 5 DEGs were upregulated across several time points, including *ADAMTS15* (single exposure: 1h, 16h), *NIN* (repeated exposure: 2d, 3d), and *P2RY11*, *CARD10*, and *ESPNP* (all time points: 1h, 16h, 2d, and 3d) (Figure 1C). The former genes (*ADAMTS15* and *NIN*) might represent distinct acutely restricted morphine-induced effects while the latter set (*P2RY11*, *CARD10*, and *ESPNP*) represents sustained morphine-induced genes. Among these, *P2RY11* showed persistent induction across all time points relative to baseline: 1h (log2FC = 1.74, *p*-adj = 1.07 × 10^−10^), 16h (log2FC = 1.63, *p*-adj = 7.81 × 10^−9^), 2d (log2FC = 1.85, *p*-adj = 2.12 × 10^−12^), and 3d (log2FC = 1.87, *p*-adj = 5.23 × 10^−13^) (Figure 1B, Supplemental Table S2).

To further explore the expression patterns of the 19 DEGs, we performed hierarchical clustering of their expression values in all samples. Notably, clustering of samples based on the expression of these DEGs cleanly grouped samples by time point as expected (Figure 1D). It also resulted in the clustering of single-exposure time points (1h, 16h) distinctly from repeated-exposure time points (2d, 3d), with the baseline samples adjacent to the repeated-exposure cluster (Figure 1D). Additionally, genes were clustered by their expression among all samples and time points (Figure 1D). The first cluster (*VRK1*, *KMT2A*, *ZNF595*, *ENSG00000281383*, *ENSG00000280800*, *ENSG00000281181*) exhibited relatively lower expression at single-exposure time points (1h, 16h) compared to the other time points (Figure 1D). The second cluster (*ASAP3*, *COPS8-DT*, *KLF9*, *ENSG00000272980*) was characterized by relatively lower expression at 3d relative to the other time points (Figure 1D). The third cluster (*ADAMTS15*, *RNASE4*, *P2RY11*, *ESPNP*, *NIN*) represents the most stably induced genes, with lowest expression at baseline (Figure 1D). Finally, the fourth cluster (*CWC27*, *CARD10*, *DCN*, *EMILIN1*) exhibited strongly induced expression at 1h (Figure 1D). This hierarchical clustering of DEG expression levels highlights the distinct transcriptomic profile of single-exposure time points vs. repeated-exposure time points and reveals key dynamic expression patterns across the time course.

Next, we performed Gene Ontology (GO)-based gene set enrichment analysis (GSEA) to understand how transcriptional profiles at each time point may translate to functional differences in nociceptors in response to morphine (Figure 1E, Supplemental Table S3). We highlighted terms enriched during at least 2 time points. The overall transcriptomic profiles of neurons at 1h and 2d demonstrated conserved enrichment of several terms, including those related to growth factor response, encapsulating structure, development, and cell migration (Figure 1E). Meanwhile, the overall profiles of neurons at 16h and 3d were generally more distinct from the other time points (Figure 1E).

### 3.2. Time-Series Differential Expression and Clustering Link P2RY11 Induction and the Dynamics of Related Genes to Opioid Exposure

**Figure 2:**
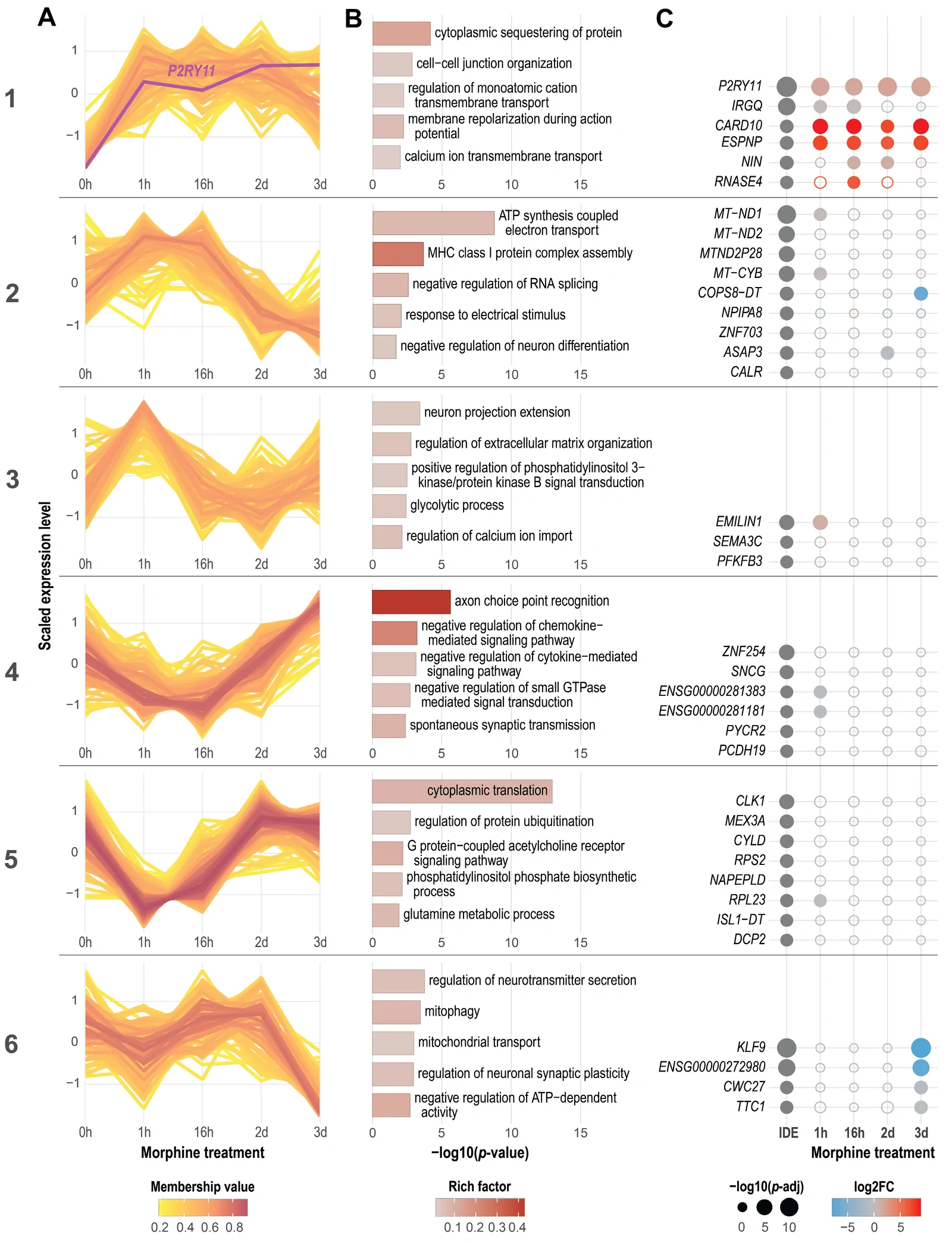
Time-series differential expression and clustering link *P2RY11* induction and the dynamics of related genes to opioid exposure. (**A**) Fuzzy c-means clustering of genes exhibiting dynamic expression over the morphine time course (ImpulseDE2 *p* ≤ 0.05), partitioned into six clusters with distinct temporal trajectories. Each line represents a gene; line color indicates membership value in the displayed cluster. (**B**) Select Gene Ontology (GO) biological process terms from over-representation analysis (ORA) for genes within each cluster. Bar length indicates enrichment significance (−log10[*p*-value]); shading indicates rich factor. (**C**) Dot plot summarizing selected dynamic DEGs (dDEGs) by cluster. The first column shows significant dDEGs from time-course analysis by ImpulseDE2 (*p*-adj ≤ 0.05), where dot size represents −log10(*p*-adj). Subsequent columns show pairwise edgeR results for each time point relative to baseline (1h, 16h, 2d, or 3d vs. 0h), where dot size indicates −log10(*p*-adj) and dot color indicates log2FC. Filled dots meet DEG thresholds (*p*-adj ≤ 0.05 and |log2FC| ≥ 0.6); outlined dots do not.

To more fully characterize the temporal transcriptional dynamics in nociceptors in response to morphine exposure, we performed time-series differential expression and clustering analyses, both of which accounted for overall longitudinal expression dynamics (rather than focusing on pairwise comparisons relative to baseline). This allowed us to identify specific patterns of coordinated gene expression dynamics over the time course (Figure 2A, Supplemental Table S4) and then profile (i) the biological processes associated with genes in each cluster (Figure 2B, Supplemental Table S4) as well as (ii) key genes included in each cluster (Figure 2C, Supplemental Table S4). ImpulseDE2 differential expression analysis identified 36 dynamic DEGs (dDEGs) whose expression level changed significantly over the time course (*p*-adj ≤ 0.05) (Figure 2C, IDE column). Genes whose expression appeared to change longitudinally (*p* ≤ 0.05 by ImpulseDE2 analysis) were included in the clustering analysis (Figure 2A).

Cluster 1 was characterized by an increase in expression that was generally sustained, at least through 2d (Figure 2A). This cluster was abundant in genes involved in cell–cell junction organization and ion flux, including cation and calcium transmembrane transport as well as membrane repolarization during action potential (Figure 2B). Notably, Cluster 1 included *P2RY11*, the strongest dDEG (*p*-adj = 1.36 × 10^−15^) and also the strongest upregulated DEG at each time point relative to baseline (by *p*-adj) (Figure 2C). Cluster 1 also included the 2 other genes in the sustained morphine-induced gene set (*CARD10*, *ESPNP*) as well as *NIN* (upregulated at 2d and 3d) (Figure 1C and Figure 2C).

Clusters 2 and 3 represented early-response genes with the highest expression at early time points (Figure 2A). Cluster 2 represented somewhat transient, early-response genes whose expression generally peaked at 1h–16h and then fell below baseline by 2d and 3d (Figure 2A). This cluster included many mitochondrial genes involved in ATP synthesis as well as genes involved in MHC class I protein complex assembly, response to electrical stimulus, and neuron differentiation (Figure 2B). Interestingly, all mitochondrial dDEGs fell into this early-response cluster (Figure 2C). Cluster 3 included highly transient, early-response genes, exhibiting peak expression at 1h that subsequently fell and fluctuated around baseline at later time points (Figure 2A). Genes involved in neuron projection extension, regulation of extracellular matrix organization, positive regulation of phosphatidylinositol 3-kinase (PI3K)/protein kinase B (PKB aka Akt) signal transduction, and calcium import fell into this cluster (Figure 2B). Cluster 3 contained the fewest dDEGs (*n* = 3).

Clusters 4 and 5 corresponded inversely with Clusters 2 and 3, including early-responding downregulated genes with the lowest expression at early time points (Figure 2A). Cluster 4 exhibited an inverse pattern to Cluster 2: a somewhat transient decrease in expression at 1h that fell until 16h, followed by increasing expression that rose to or above baseline by 3d (Figure 2A). Genes in this cluster were involved in axon choice point recognition, spontaneous synaptic transmission, and negative regulation of cytokine- or small GTPase-mediated signaling (Figure 2B). Meanwhile, Cluster 5 exhibited a roughly inverse pattern to Cluster 3, with lowest expression at 1h, followed by an increase to and stabilization around or slightly above baseline at 2d and 3d (Figure 2A). This cluster included genes involved in translation, protein ubiquitination, GPCR signaling, and phosphatidylinositol phosphate (PIP) biosynthesis, for example (Figure 2B). Finally, Cluster 6 represented genes whose expression steeply fell between 2d and 3d, with fluctuation at earlier time points (Figure 2A). Cluster 6 included genes related to neurotransmitter secretion, mitochondrial transport, and negative regulation of neuronal synaptic plasticity or ATP-dependent activity (Figure 2B).

Altogether, the time-series differential expression analysis and clustering analyses point to the potential of *P2RY11* and P2Y11-mediated signaling as a candidate morphine-responsive pathway in the opioid response of human nociceptors, warranting direct functional investigation. For example, *P2RY11* was identified as a highly significant DEG across time points and analytical approaches. Additionally, other genes associated with processes both upstream (ATP generation, mitochondrial function) and downstream (GPCR signaling, PIP-mediated signaling, modulation of calcium flux, cytokine signaling) of P2Y11-mediated signaling exhibit coordinated dynamic expression along the time course. We emphasize that these enrichment and clustering analyses reflect coordinated transcriptional correlations and are hypothesis-generating; they do not demonstrate that P2Y11 signaling controls these pathways, which will require direct functional testing. Among the sustained morphine-induced genes, we prioritized *P2RY11* because it uniquely combines the strongest and most persistent induction, human specificity (no mouse or rat ortholog), and druggable GPCR biology; other candidates (*CARD10* or *ESPNP*) remain candidates for future study.

### 3.3. P2RY11/P2Y11 Is Expressed in hDRG Neurons and Increases with Morphine Exposure

**Figure 3:**
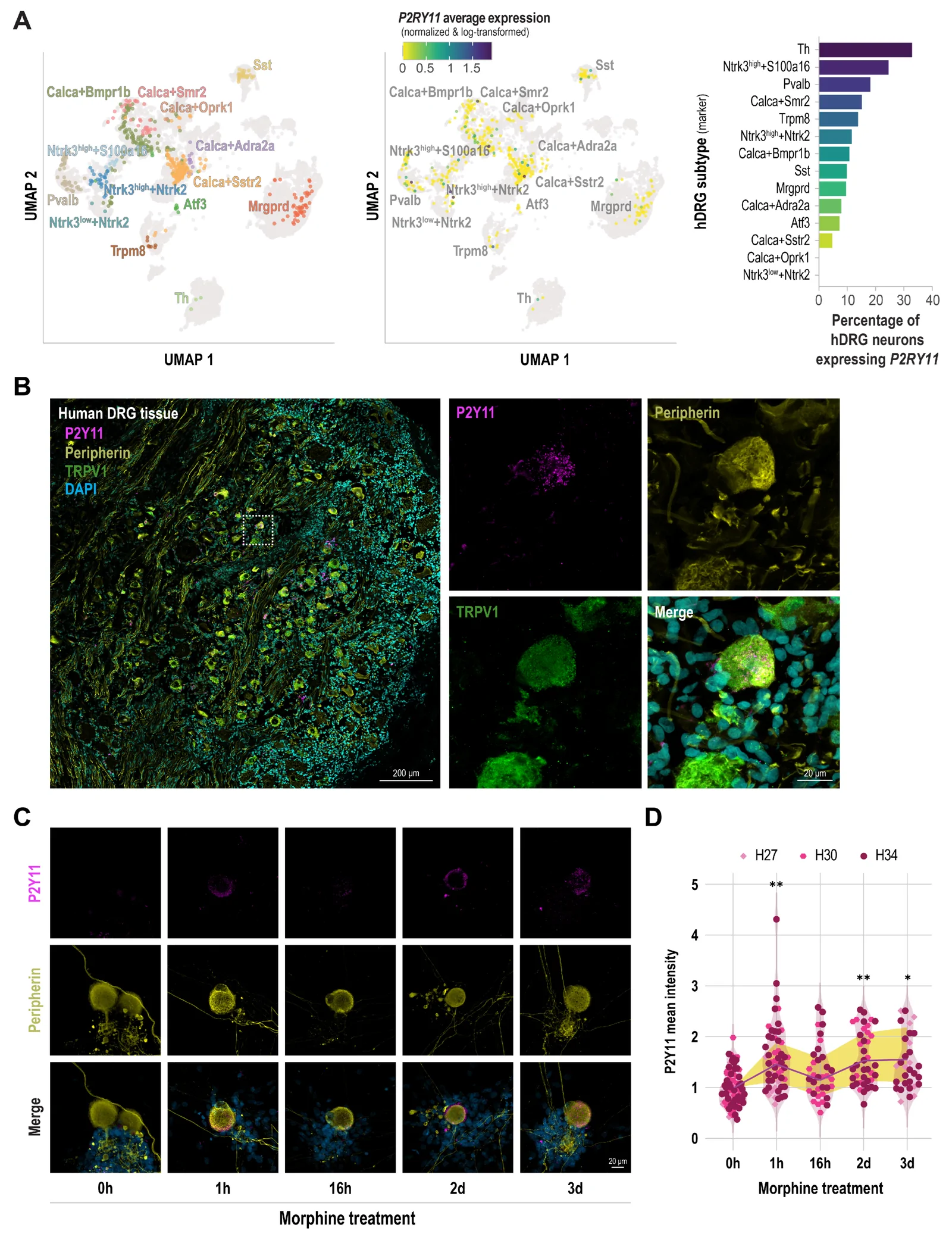
*P2RY11*/P2Y11 is expressed in hDRG neurons and increases with morphine exposure. (**A**) *P2RY11* expression across human dorsal root ganglion (hDRG) neuronal subtypes using harmonized human single-nucleus DRG datasets. Left, UMAP of hDRG neuronal subtypes; middle, feature plot showing average *P2RY11* expression (normalized and log-transformed); right, bar plot showing the percentage of neurons expressing *P2RY11* within each subtype. (**B**) Representative immunofluorescence images of human DRG tissue showing P2Y11 co-localization with peripherin and TRPV1, supporting expression in nociceptor populations. Right panels show higher-magnification views of the boxed region. Scale bars: 200 μm (left) and 20 μm (right). (**C**) Representative immunocytochemistry images of primary cultured hDRG neurons from three independent organ donors treated with morphine (3.5 μM) for the indicated durations (0h, 1h, 16h, 2d, 3d) and stained for P2Y11 and peripherin (merge shown with DAPI). (**D**) Quantification shows P2Y11 background-adjusted mean intensity normalized to the mean 0h intensity of the corresponding donor. Each small symbol represents a peripherin-positive neuronal ROI, with symbol shape and color indicating donor (239 neuronal ROIs from 129 imaging fields and 14 cultures across 3 donors). The purple line and yellow shading connect the fold-changes and reflect 95% confidence intervals, respectively, back-transformed from a linear mixed-effects model fit to log2-transformed, background-adjusted P2Y11 mean grey values, with time point as a fixed effect and donor, culture/coverslip, then imaging field as nested random effects. P2Y11 immunoreactivity was significantly increased at three morphine exposure time points relative to baseline (Dunnett-adjusted; * *p* < 0.05, ** *p* < 0.01).

Given the potential significance of *P2RY11*/P2Y11 in opioid response, we next sought to characterize its expression in human nociceptors, including after morphine exposure. We evaluated its expression in hDRG, including in tissue-derived single-nucleus transcriptomic datasets, in tissue sections, and in cultured cells. First, we explored *P2RY11* expression in hDRG neuron subtypes utilizing a resource of harmonized single-cell RNA-seq data from the DRG of several rodent and primate species that classified pan-species DRG subtypes and their gene expression profiles [39]. Within the confines of pan-species clustering and subtype classification, we specifically referenced subtype-specific *P2RY11* expression levels from 3 single-nucleus hDRG studies (Figure 3A). *P2RY11* expression was detected across most hDRG neuron subtypes rather than restricted to a single class (Figure 3A). The percentage of *P2RY11*-positive neurons per subtype varied from 0–33.3%, with the greatest proportions in Th (33.3%), Ntrk3+S100a16 (25.0%), Pvalb (18.6%), Calca+Smr2 (15.6%), and Trpm8 (14.3%) subtypes (Figure 3A). 

We next validated the presence and expression of P2Y11 in hDRG tissue. We detected P2Y11 signal in peripherin-positive sensory neurons, including TRPV1-positive nociceptor populations (Figure 3B). Furthermore, in cultured hDRG neurons from three independent organ donors, P2Y11 immunoreactivity was detectable at baseline in peripherin-positive neurons (Figure 3C,D). In alignment with *P2RY11* upregulation after morphine exposure in the time-series transcriptomic dataset (Figure 1 and Figure 2), semiquantitative analysis of P2Y11 immunoreactivity in peripherin-positive hDRG neurons revealed higher P2Y11 intensity at most morphine exposure time points relative to baseline: 1h (fold-change [FC] 1.46, 95% CI 1.121–1.89, *p* = 0.0036), 16h (FC 1.15, 95% CI 0.836–1.58, *p* = 0.5823), 2d (FC 1.53, 95% CI 1.137–2.07, *p* = 0.0046), and 3d (FC 1.55, 95% CI 1.104–2.18, *p* = 0.0108) (Figure 3D). These estimates were reproduced when adjusted mean grey values from the culture/coverslip rather than individual neuronal ROIs were used as the unit of observation (FC 1.48, 1.21, 1.47, and 1.57 at 1h, 16h, 2d, and 3d, respectively). P2Y11 induction was consistent in direction across all donors, with all morphine-treated cultures exceeding the corresponding donor baseline (sign test *p* = 0.0010), and the increase remained significant when each donor was reduced to a single value (FC 1.43, 95% CI 1.13–1.83, *p* = 0.024). Collectively, these results demonstrate that *P2RY11* is an opioid-responsive transcript in hiPSC-derived nociceptors and is expressed across many hDRG neuron subtypes. Additionally, P2Y11 expression is detectable in ex vivo human nociceptors and induced in hDRG neurons by morphine exposure in vitro.

## 4. Discussion

In the present study, we used hiPSC-derived nociceptors to identify opioid-responsive gene programs and discovered *P2RY11* as a robust morphine-inducible transcript. Across time points and analyses, regulation of purinergic signaling-related genes—particularly induction of *P2RY11*/P2Y11 expression—was among the most prominent results of morphine exposure. *P2RY11* exhibited sustained upregulation in our longitudinal dataset, and genes in pathways related to P2Y11 and purinergic signaling (e.g., ATP synthesis, GPCR signaling, calcium ion flux, and cytokine signaling) exhibited coordinated expression dynamics. To further establish human relevance, we extended these findings to hDRG tissue and primary DRG cultures from organ donors. *P2RY11* transcripts were detected across hDRG neuronal subtypes in available single-nucleus transcriptomic datasets, in line with published single-nucleus and spatial transcriptomic surveys of human DRG [6,8]. In parallel, we detected P2Y11 protein in peripherin- and TRPV1-positive hDRG neurons, providing ex vivo evidence that P2Y11 is present in nociceptor populations. Furthermore, we found that P2Y11 immunoreactivity increases after morphine treatment, consistent with our sequencing results. These data nominate *P2RY11*/P2Y11 as an opioid-responsive purinergic receptor in human nociceptors and provide a foundation for defining its potential contribution to opioid-driven peripheral plasticity. We interpret the morphine-induced changes reported here as early transcriptional events in human nociceptors that may precede and prime longer-term plasticity rather than as correlates of established tolerance or OIH, which develop over weeks to months. Accordingly, we regard *P2RY11*/P2Y11 as a morphine-responsive candidate pathway whose functional contribution remains to be tested.

Prolonged opioid treatment has been shown to engage compensatory signaling and neuropeptide programs that are mechanistically linked to tolerance and paradoxical sensitization. In cultured DRG neurons, sustained morphine exposure in the low-micromolar range augments basal and evoked CGRP release and is associated with adenylyl cyclase superactivation/cAMP overshoot, whereas longer paradigms increase CGRP and substance P immunoreactivity, consistent with durable remodeling of peptidergic nociceptor programs [25,26,41]. Guided by this literature, we used a 3.5 μM morphine paradigm to maintain sustained MOR-1 engagement in a simplified human nociceptor preparation and to capture transcriptional programs that emerge with prolonged exposure. However, these in vitro exposure paradigms should not be interpreted as direct recreations of human morphine pharmacokinetics. In patients, morphine exposure is route- and regimen-dependent; for example, mean plasma concentrations around 21 ± 12 ng/mL have been reported during postoperative intravenous patient-controlled analgesia, whereas mean steady-state concentrations during chronic oral therapy in cancer patients ranged from 5.9 to 68.4 ng/mL [42,43]. In addition, morphine is only partially protein-bound in plasma (34.0–37.5%), and clinically relevant exposure includes compartmental distribution and the formation of morphine-3-glucuronide (M3G) and morphine-6-glucuronide (M6G), which are not modeled in a simple parent-drug culture system [44,45]. Route of administration further shapes metabolite exposure; patients receiving oral morphine have higher glucuronide/morphine ratios than those receiving subcutaneous therapy, consistent with first-pass glucuronidation [46,47]. Taken together, our in vitro design is best viewed as a mechanistically informed model of sustained parent-morphine signaling in human nociceptors rather than a pharmacokinetic surrogate for any single clinical dosing regimen.

To place our findings in a cross-species context, we compared our human nociceptor response to rodent data at three levels (Supplemental Table S5). Because no rodent morphine-treated DRG transcriptomic dataset comparable to ours exists, a direct differential-expression overlap was not possible, a gap that itself reflects the human-focused rationale of our study. First, we mapped 42 morphine-responsive genes (DEGs and dDEGs, which includes overlapping genes) to mouse and rat orthologs using Ensembl BioMart, with every gene not classified as having one-to-one orthologs verified manually in Ensembl Compara and NCBI Gene. Thirty (71%) had one-to-one orthologs in both species, whereas 12 (29%) had no rodent ortholog, comprising five protein-coding genes (including *P2RY11*), five lncRNAs, and two pseudogenes. Thus, *P2RY11* is not an isolated exception. Second, for genes with conserved orthologs, we compared expression across human, mouse, and rat DRG neuronal subtypes using the harmonized cross-species DRG atlas [39]. Of the 30 conserved genes, 29 were detectable in both human and mouse DRG neurons; *RPS2* could not be assessed because the atlas feature mapping inherited the incomplete Ensembl ortholog assignment (Supplemental Table S5). Because the contributing datasets differ in platform, sequencing depth, and neuronal sampling, we limit this comparison to presence versus absence and do not interpret it as a quantitative comparison of expression levels between species. These data therefore speak to the presence of these genes in rodent models rather than to whether the opioid response itself diverges across species; establishing the latter would require a directly comparable morphine-treated rodent DRG dataset, which does not currently exist to the best of our knowledge. Third, at the mechanistic level, our response converges with rodent opioid models on GPCR, calcium, cAMP/PKA, and MAPK/CREB signaling and on purinergic involvement. Notably, rodent purinergic contributions to tolerance and OIH act largely through microglial P2X4 and P2X7 signaling [17,18,19,48], whereas our data implicate a neuron-intrinsic, human-specific P2Y-family receptor. Together, these analyses indicate that species differences in the opioid response of sensory neurons may arise principally from the outright absence of orthologs rather than from divergent expression of conserved genes. They position *P2RY11* as one example of a broader class of human-relevant opioid-responsive pathways not represented in standard rodent models.

Our central finding is that morphine exposure increases expression of *P2RY11* transcript and P2Y11 protein in human nociceptors. Purinergic signaling is a core component of nociceptor physiology: extracellular nucleotides, particularly ATP, are released during tissue injury, inflammation, and cellular stress and engage P2X ionotropic channels and P2Y GPCRs on neurons and non-neuronal cells along the pain axis [49,50,51]. Our findings extend this purinergic–opioid framework by identifying a P2Y-family GPCR that is transcriptionally induced by morphine within human nociceptors. P2Y11 is distinctive within the P2Y family because it couples to both phospholipase C/calcium signaling and adenylyl cyclase/cAMP signaling [52,53]. Across human immune and vascular cell types, including monocyte-derived dendritic cells, macrophages, CD4 T cells, and endothelial cells, P2Y11 activation reshapes ATP-driven second-messenger signaling and influences cell-state transitions and motility [54,55,56,57,58]. Notably, these effects are context-dependent rather than uniformly pro-inflammatory, encompassing both inflammatory programs—such as IL-6 production and M1-like polarization—and anti-inflammatory, cytoprotective, and pro-angiogenic programs—such as IL-10-driven M2 polarization and delayed apoptosis [21,22,55,59,60,61,62]. Extrapolated cautiously to nociceptors, these observations suggest two non-exclusive models: (i) morphine-induced *P2RY11*/P2Y11 upregulation may increase the gain of ATP-evoked signaling and thereby amplify plasticity relevant to tolerance or OIH or (ii) it may represent an adaptive or homeostatic response to opioid-induced cellular stress. Direct functional experiments will be required to distinguish between these possibilities.

Our findings also underscore a broader translational issue: *P2RY11* lacks a murine ortholog, which limits conventional rodent genetic approaches and likely contributes to the relative scarcity of pain-focused studies on this receptor [20]. This absence complicates interpretation of “P2Y11-like” pharmacology reported in rodents, because agonists and antagonists often have cross-selectivity across P2Y receptors and may engage other targets when the canonical receptor is not present [20,63]. In this context, human-based platforms are not simply confirmatory but are essential for discovery. By combining a controlled morphine exposure paradigm in hiPSC-derived nociceptors with validation in hDRG tissue and primary cultures, we establish both inducibility and human relevance, thereby addressing an important translational gap. Species differences in pain biology, the limitations of rodent models, and the value of human transcriptomic and sensory-neuron approaches are emphasized in recent work [64,65,66,67].

Several limitations should be considered. First, although we identify *P2RY11*/P2Y11 as an opioid-responsive transcript/protein, we do not establish causality, functional consequences, or whether P2Y11 contributes to analgesia, tolerance, or OIH. Increased expression could plausibly enhance ATP-evoked calcium and cAMP signaling, but the context-dependent literature above leaves open that it instead reflects an adaptive or homeostatic response. Second, our transcriptomic discovery used *n* = 3 independent cultures per time point, reasonable for exploratory time-series RNA-seq in a homogeneous cell culture but limited in power. Thus, the resulting DEGs should be regarded as a conservative, high-confidence set rather than an exhaustive catalog. Third, because the repeated-exposure paradigm includes washout periods, it may model intermittent dosing but could also introduce withdrawal-like components. Since cAMP overshoot can occur upon opioid removal, disentangling sustained MOR-1 activation from withdrawal-driven effects will be important. Finally, our human validation relied on a single commercial hiPSC-nociceptor line from one donor and on primary hDRG cultures from a limited number of organ donors, and P2Y11 protein was detected with a single antibody. Although supported by transcript-level detection in our RNA-seq and in independent single-nucleus datasets, definitive protein specificity would benefit from orthogonal validation such as peptide competition, a second antibody, RNAscope, and/or knockdown.

These limitations define clear next steps. Comparing continuous versus intermittent exposure paradigms and incorporating MOR-1 antagonism or genetic suppression during exposure will help separate effects of sustained MOR-1 activation from withdrawal-associated adaptations. Functional testing of *P2RY11* using CRISPR interference or antisense oligonucleotides, ideally paired with cautious pharmacology-given ligand selectivity constraints across P2Y receptors [20,63], will determine whether P2Y11 is required for morphine-induced transcriptional dynamics and/or changes in excitability. Mechanistic assays of intracellular calcium and cAMP signaling can test whether morphine exposure increases ATP-evoked second-messenger responses in a P2Y11-dependent manner, and measurements of extracellular nucleotide release can clarify whether opioids reshape the ATP milieu. Finally, extending these analyses to additional opioids and leveraging single-cell approaches to resolve subtype- or state-specific regulation, together with integration of donor metadata and exposure histories when available, will define the broader clinical scope of *P2RY11* regulation.

In summary, our time-series transcriptomic analysis of hiPSC-derived human nociceptors identifies *P2RY11* as a robust morphine-inducible gene. Together with human DRG transcriptomic, protein localization, and primary DRG culture validation, this establishes P2Y11 as a previously underappreciated component of opioid-responsive signaling in human sensory neurons. These results expand the set of human-relevant opioid-responsive targets and highlight the importance of human-based models for uncovering pain mechanisms.

## Data Availability

RNA-seq data generated in this study have been deposited in NCBI’s Sequence Read Archive (SRA) under BioProject accession PRJNA1505724. accessed on 10 August 2026Results from the differential expression, clustering, and enrichment analyses are available in the Supplemental Tables.

## Supplementary Materials

The following supporting information can be downloaded at: https://www.mdpi.com/article/10.3390/cells15161464/s1. Supplemental Figure S1: Repeated morphine exposure paradigm increases mu-type opioid receptor (MOR-1) expression in hiPSC-derived nociceptors; Supplemental Figure S2: Antibody testing in human DRG cultures confirms specificity of secondary antibodies for the respective P2Y11 or peripherin primary antibody; Supplemental Table S1: Human DRG donor demographics and sample allocation; Supplemental Table S2: Pairwise differential gene expression analysis at each morphine time point relative to baseline; Supplemental Table S3: Pairwise gene set enrichment analysis (GSEA) results for each morphine time point relative to baseline; Supplemental Table S4: Time-series differential expression (ImpulseDE2) and clustering analysis results; Supplemental Table S5: Cross-species conservation and DRG expression of morphine-responsive genes.

## Abbreviations

DRG	dorsal root ganglion
hDRG	human dorsal root ganglion
hiPSC	human induced pluripotent stem cell
MOR-1	mu-type opioid receptor
OIH	opioid-induced hyperalgesia
DEG	differentially expressed gene
dDEG	dynamic differentially expressed gene
GPCR	G protein-coupled receptor
ICC	immunocytochemistry
IHC	immunohistochemistry
ROI	region of interest
GO	Gene Ontology
GSEA	gene set enrichment analysis
ORA	over-representation analysis
CGRP	calcitonin gene-related peptide
