# Supplementary material for: Opioid Exposure Induces the Expression of the Purinergic Receptor P2RY11 in Human Nociceptors
Source: Cells. 2026 Aug 15;15(16):1464. doi: 10.3390/cells15161464 (PMC13511635; doi:10.3390/cells15161464)
Supplement: Supplementary file 1 [file cells-15-01464-s001.zip › Supplemental Figure S1.pdf]

Supplemental Figure S1
Demeter et al., *Cells*, 2026

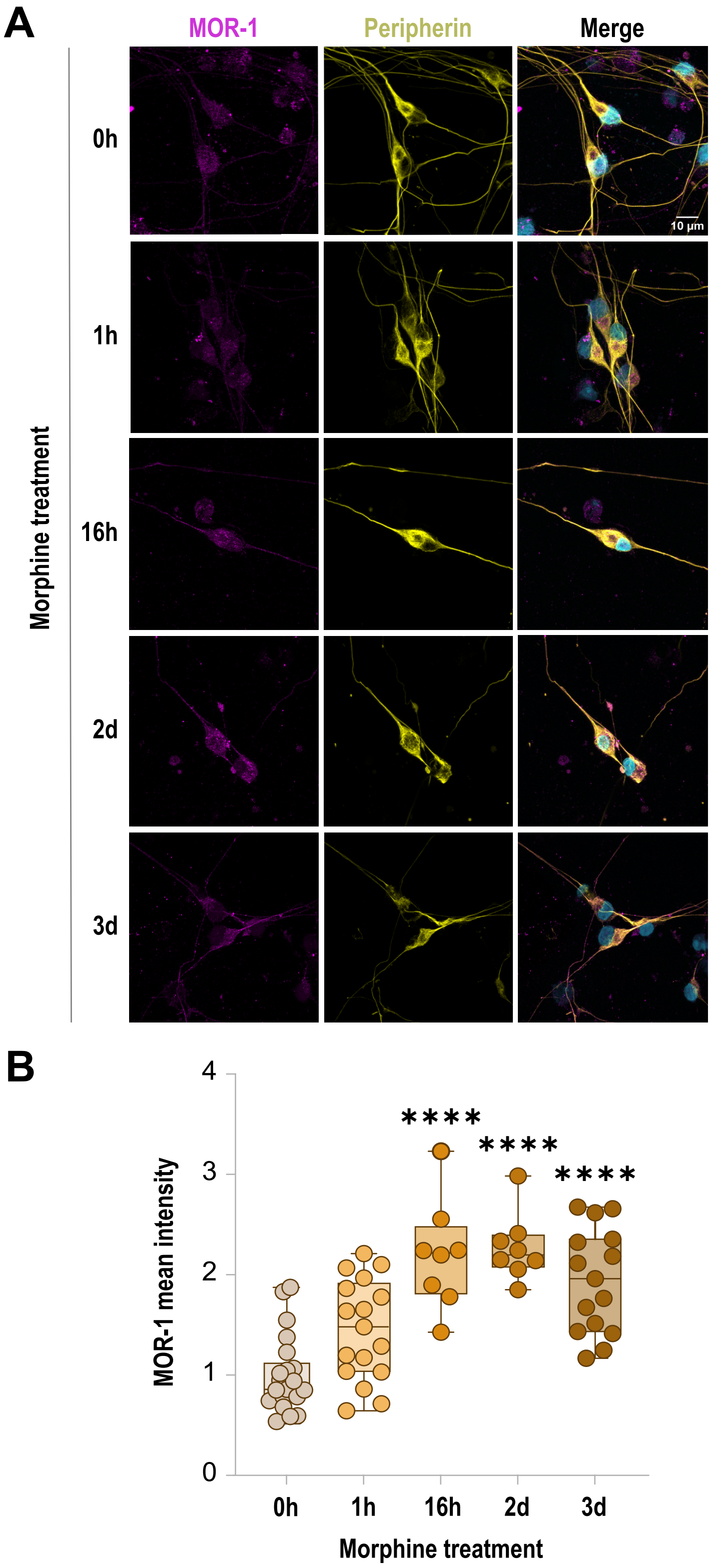

Supplemental Figure S1. Repeated morphine exposure paradigm increases mu-type opioid receptor (MOR-1) expression in hiPSC-derived nociceptors.
(**A**) Representative immunocytochemistry images showing peripherin and MOR-1 immunoreactivity across time points, confirming a nociceptor-like phenotype and sustained MOR-1 signal following morphine exposure. Merge includes MOR-1, peripherin, and DAPI. Scale bar: 10 µm.
(**B**) Quantification of MOR-1 adjusted (background-subtracted) mean fluorescence intensity measured in peripherin-defined ROIs and normalized to 0h controls. Each dot represents a single peripherin-positive ROI (cell or cluster). Asterisks indicate significance relative to 0h (**** $p < 0.0001$, Kruskal-Wallis one-way ANOVA test, followed by Dunn's test).
