# Supplementary material for: Opioid Exposure Induces the Expression of the Purinergic Receptor P2RY11 in Human Nociceptors
Source: Cells. 2026 Aug 15;15(16):1464. doi: 10.3390/cells15161464 (PMC13511635; doi:10.3390/cells15161464)
Supplement: Supplementary file 1 [file cells-15-01464-s001.zip › Supplemental Figure S2.pdf]

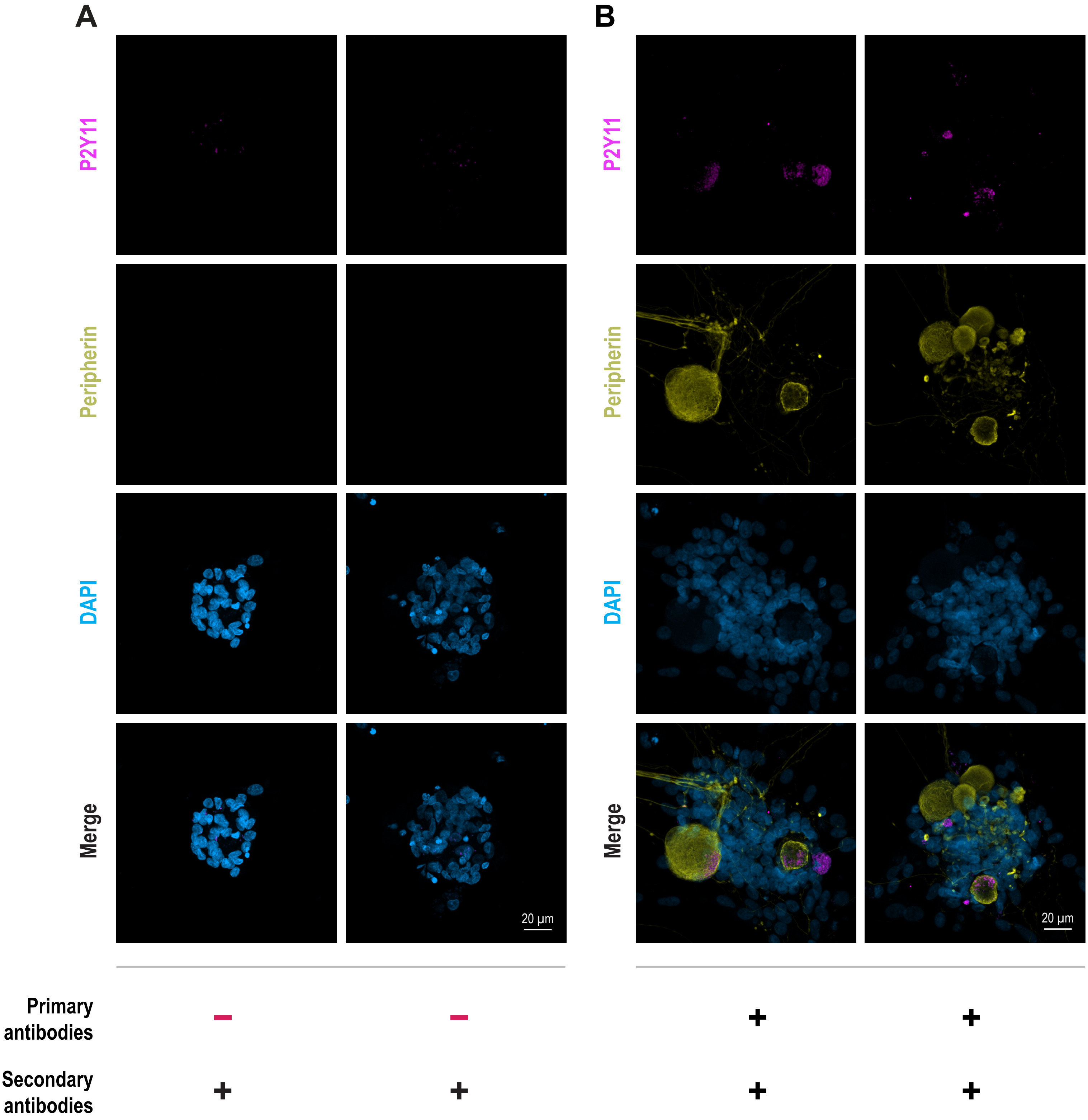


**Supplemental Figure S2. Antibody testing in human DRG cultures confirms specificity of secondary antibodies for the respective P2Y11 or peripherin primary antibody.**
(**A**) Representative immunocytochemistry images of human DRG cultures showing minimal secondary antibody signal in cells incubated with no primary antibodies, only secondary antibodies.
(**B**) Representative immunocytochemistry images of human DRG cultures showing both P2Y11 and peripherin immunoreactivity in cells incubated with both primary and secondary antibodies.
Images in (**A**) and (**B**) were acquired and processed using the same settings.
